# *Candida albicans* Enhances Protease Activity and Activates MyD88‐Dependent IL‐1β Production in Human Keratinocytes

**DOI:** 10.1111/myc.70133

**Published:** 2025-11-26

**Authors:** Jingyi Wang, Neil A. R. Gow, Matthew G. Brewer

**Affiliations:** ^1^ Department of Dermatology University of Rochester Rochester New York USA; ^2^ Medical Research Council Centre for Medical Mycology, University of Exeter Exeter UK

**Keywords:** atopic dermatitis, Candida, IL‐1β, keratinocyte, MMP‐9, mycobiome, MyD88, protease

## Abstract

**Background:**

Atopic dermatitis (AD) is a common chronic skin disorder characterised by a highly inflamed local environment and elevated epidermal proteolytic activity. Changes in the skin mycobiome have been observed in this disease, specifically *Candida albicans* colonization positively correlating with AD severity, yet the mechanisms by which this fungus contributes to disease features remain elusive.

**Objectives:**

This study aimed to elucidate how *C. albicans* can influence AD pathogenesis through its influence on keratinocyte (KC) proteolytic activity, inflammatory cytokine secretion and epidermal barrier integrity, as well as define the signaling pathways mediating these effects.

**Methods:**

Immortalized human KC were co‐cultured with *C. albicans* and changes in KC protease expression and activity, along with the secretion of the pro‐inflammatory cytokine IL‐1β were assessed. Additionally, the impact of IL‐1β on KC barrier formation was determined using transepithelial electrical resistance. To identify signalling pathways mediating *Candida*‐induced phenotypes, CRISPR/Cas9 was used to establish cell lines deficient in myeloid differentiation primary response protein 88 (MyD88) or matrix metalloprotease‐9 (MMP‐9).

**Results:**

*C. albicans* induced proteolytic activity from KC through fungal secreted aspartyl proteases (Sap4‐6) and promoted IL‐1β secretion via MyD88 signalling. This response increased expression and activation of host MMP‐9 and led to impaired barrier function. Genetic deletion of either *MYD88* or *MMP9* restored barrier function in IL‐1β treated cells, suggesting MMP‐9 serves as a downstream effector of IL‐1β/MyD88 signalling.

**Conclusion:**

These findings establish a mechanistic link between skin resident fungi and epidermal barrier dysfunction. We demonstrate a pathway linking fungal colonization to innate immune responses by skin cells, providing insight into how the commensal fungus *C. albicans* may contribute to AD pathogenesis.

## 1 Introduction

Fungal infections of the skin induce a complicated and multifaceted host response. To dissect the unique interplay between fungal colonization and skin homeostasis, we investigated how a key signalling protein in innate immune responses, myeloid differentiation primary response protein 88 (MyD88), modulates fungal‐induced epidermal inflammation. The human epidermis is a complex, stratified tissue comprised of multiple layers, each contributing distinct functions essential for maintaining epithelial integrity and defending against external pathogens. Keratinocytes (KC), the most abundant cell type in the epidermis [1], sustain this protective structure through continuous proliferation and differentiation. During differentiation, KC develop two sequential barrier structures: tight junctions and the stratum corneum [1]. Tight junctions, located beneath the stratum corneum within the stratum granulosum, regulate the flux of ions, water, and macromolecules both into and out of the skin [2]. Additionally, the stratum corneum, consisting of terminally differentiated KC, forms an impermeable, keratin‐rich shield [3]. Disruption of these two barriers is associated with cutaneous inflammatory diseases, such as atopic dermatitis (AD) [4]. Therefore, understanding how different components of the skin, including the cutaneous microbiome, affect barrier function is an ongoing focus in fostering skin health.

A hallmark of AD is an altered skin microbiome [5]. While *Staphylococcus aureus* has been extensively studied for its role in AD pathogenesis [6, 7], contributions from skin‐resident fungi are less well understood. Previous research has highlighted a link between *Malassezia* colonization and AD‐like disease [8, 9], with one key finding being the positive association of *Malassezia*‐specific IgE antibodies and AD [ 10]. Supporting this, patients with severe AD exhibit higher sensitization to *Malassezia* than those with mild‐to‐moderate disease phenotypes [11]. However, the role of other fungal species in AD pathophysiology remains underexplored. Recent microbial sequencing analyses of skin samples revealed a significantly higher prevalence of *Candida albicans* in AD patients compared to healthy controls [12, 13, 14]. Additionally, a positive correlation has been observed between AD severity and *C. albicans* ‐specific IgE antibodies, which were detected using the m5 *C. albicans* extract containing major allergenic components of the fungus [11]. Together, these findings suggest that immune responses triggered by *C. albicans* may contribute to AD pathogenesis. However, the mechanisms through which *C. albicans* disrupts skin homeostasis require further investigation.

While the microbiome has been associated with changes in epidermal barrier function [15], another important factor in barrier regulation is the maintenance of protease activity. Endogenous proteases are distributed across the epidermal layers and involved in various processes such as cornification [16], desquamation [17], and pro‐inflammatory signalling [18]. Beyond host‐derived proteases, microbial‐secreted proteases also alter epidermal function. For example, proteases from *S. aureus* increase trypsin activity in primary KC, facilitating filaggrin degradation [19]. Likewise, *C. albicans* produces multiple secreted aspartyl proteinases (Saps) that activate protease‐activated receptor 2 on oral epithelial cells, leading to CXCL8 release and barrier impairment [20]. Additionally, dysregulation of protease activity has been implicated in the progression of various cutaneous diseases. For instance, elevated expression of kallikrein‐related peptidase 7 (KLK7), the most abundantly expressed KLK in AD skin, is linked to pathology in both AD and Netherton syndrome [21, 22, 23]. Similarly, increased levels of matrix metalloproteinase‐9 (MMP‐9) are associated with AD and bullous pemphigoid [24, 25, 26]. Among the various MMP family members, MMP‐9 shows prominent activity during acute AD flares and is elevated in response to IL‐13, a key cytokine driving AD‐associated inflammation [24, 27]. Therefore, strict regulation of epidermal protease activity is essential for maintaining skin health and identifying factors that disrupt this balance is critical for understanding disease pathogenesis.

While the importance of proteolytic activity for normal skin function is thoroughly established, how *C. albicans* influences this regulation, and whether its own Saps are key mediators remains largely unknown. To examine the impact of *C. albicans* on AD‐like phenotypes, we exposed immortalized human N/TERT‐2G KC to *Candida* species and measured changes in KC protease expression, total proteolytic activity, as well as secretion of the pro‐inflammatory cytokine IL‐1β. We then assessed the impact of IL‐1β on protease expression and barrier function. Finally, we evaluated the role of MyD88 signalling and MMP‐9 activity in driving *C. albicans* ‐induced phenotypes. Our findings reveal a novel mechanism by which skin‐resident fungi can impair barrier function through the coordination of inflammatory and proteolytic pathways.

## 2 Materials and Methods

### 2.1 Fungal Strains and Culture Conditions

The *Candida albicans* Sap‐deficient strains used in this study were previously generated in the CAI4 background (a congenic derivative of the wild‐type SC5314 strain [28]) through sequential gene disruption via homologous recombination. Both alleles of each target *sap* gene were replaced using a recyclable *hisG‐URA3‐hisG* cassette, as previously described [29]. The final triple *sap*‐deficient mutant strains retain *URA3* at a single *sap* locus to maintain prototrophy, while other disruptions were resolved through 5‐FOA–mediated marker excision [29, 30]. The *ece1*∆/∆ strain used in this study was generated in the BWP17 background (an auxotrophic derivative of SC5314 [28]), of which sequential transformation was accomplished via the *ece1*‐*his1* and *ece1*‐*arg4* deletion cassettes, followed by reintroduction of the CIp10 vector [31]. Clinical isolates of *C. albicans* , *C. tropicalis* , *C. parapsilosis* , and *C. glabrata* (now known as *Nakaseomyces glabratus*) were provided by Dr. Andrew Cameron (Clinical Microbiology Laboratories, Department of Pathology and Laboratory Medicine, University of Rochester, Rochester) and through the University of Exeter (Table S1). A single colony from each strain was inoculated into Yeast‐Peptone‐Dextrose broth (Cat # Y1375, Millipore Sigma) and incubated overnight at 37°C. This was used to establish a glycerol stock for long‐term storage at −80°C. For co‐culture experiments with KC, an individual *Candida* colony was inoculated into 5 mL of YPD broth and incubated at 37°C with shaking at 400 rpm for 18 h. After incubation, yeast cell density was measured by OD600 values and cell abundance (colony forming units [CFU]/mL) was calculated using a previously established growth curve.

### 2.2 Cells and Culture Techniques

The immortalized KC cell line N/TERT‐2G was provided by Dr. Ellen H. van den Bogaard (Department of Dermatology, Radboud University, Nijmegen, Netherlands) and propagated as previously described [32, 33]. N/TERT‐2G cells were maintained in keratinocyte serum‐free media (KSFM) 1X (Cat # 17005042, Gibco) supplemented with bovine pituitary extract, epidermal growth factor, antimicrobials (penicillin/streptomycin, amphotericin B (Gibco) and 0.3 mM CaCl_2_ (Cat # MT‐140, Boston Bioproducts)). Cells were expanded to approximately 30% confluency, then trypsinized and plated into 24‐well plates at a density of 1.5 × 10^5^ cells per well in 500 μL KSFM. Following a 48 h proliferation period, differentiation was induced by replacing KSFM with Dulbecco's Modified Eagle Medium ([DMEM] Cat # 21068, Gibco) supplemented with 4 mM L‐glutamine (Cat # 25030149, Gibco) and 1.8 mM CaCl_2_. Treatments including 10^3^ CFU of yeast or different concentrations (10, 100, or 1000 pg/mL) of recombinant IL‐1β (Cat # 201‐LB‐005/CF, R&D Systems) were introduced 48 h post‐differentiation. After 24 h of exposure, KC were harvested for subsequent analyses.

### 2.3 CRISPR/Cas9‐ Based Gene Editing in Immortalised KC

A CRISPR/Cas9 knockout system targeting *MYD88* and *MMP9* was used according to the manufacturer's instructions (Gene KnockoutKit v2, EditCo). Each kit comprised recombinant Cas9 protein and three single‐guide RNAs (sgRNAs) with the following sequences for *MYD88*: (1) UCCUGGAGCCUCAGCGCGGU, (2) GGAGGAUGUGGAGGAGACCG and (3) GUUCUUGAACGUGCGGACAC, and *MMP9*: (1) CAGCUCACCGGUCUCGGGCA, (2) CAGGAAUACCUGUACCGCUA and (3) GUCUGGGACCCCGCACCGUG. The Cas9 ribonucleoprotein complex was assembled with the sgRNAs according to the manufacturer's instructions. Electroporation‐based cell transfection was performed using the Neon Transfection System (ThermoFisher Scientific) following previously established methods [34]. This methodology induces double‐stranded DNA breaks at each of the targeted loci, which causes large deletions in the targeted gene sequence. After electroporation, cells were transferred to 6‐well plates containing pre‐warmed culture medium for subsequent expansion.

### 2.4 Genomic DNA Isolation, PCR and Gel Electrophoresis for Knockout Validation

Following CRISPR/Cas9 editing, cells were expanded from 6‐well plates to 25 cm^2^ tissue culture flasks. Genomic DNA was isolated from 10^6^ cells using PureLink Genomic DNA Mini Kit (Cat # K182001, Invitrogen) and resuspended in 50 μL of nuclease‐free water. DNA concentration was determined by nanodrop using a SpectraDrop Micro‐Volume Microplate (Molecular Devices). Subsequently, PCR was used to amplify the edited region of the gene of interest. Each 20 μL PCR mix consisted of 6 μL nuclease‐free water, 10 μL Accustart (Cat # 95137, Quantabio), 2 μL primer mix for either *MYD88*: Forward‐TTCCCATACCCCTACTGGCA and Reverse‐GGCAGGAAATGGGGTCTCTC (933 bp insert) or *MMP9*: Forward‐AGTGGGCTGATACCGTCTCT and Reverse‐GGACACCCCATATCGCAGAG (695 bp insert) at 10 μM and 2 μL of gDNA at 25 ng/μL. PCR amplification of *MYD88* and *MMP9* was done using a SimpliAmp Thermal Cycler (ThermoFisher Scientific) under the following conditions: initial denaturation at 95°C for 10 min, followed by 30 cycles of amplification (95°C for 30 s, annealing at 58°C for *MYD88* or 59°C for *MMP9* for 30 s, 72°C for 1 min), and a final extension at 72°C for 7 min. 10 μL of PCR product was separated on a 2% agarose 6 cm gel containing GelRed Nucleic Acid GelStain (Cat # 41003, Biotium) at 125 V for 50 min. Gels were imaged with the BioRad Gel Doc XR Imaging System (Cat # 170–8195, Bio‐Rad) to visualize successful editing of the targeted gene. The sizes of PCR products were confirmed using a 100 bp ladder (Cat # N3231S, NEW ENGLAND Biolabs).

### 2.5 Transepithelial Electrical Resistance (TEER)

Barrier function of monolayer culture was assessed by TEER as previously described [35]. KC were plated at a density of 0.75 × 10^5^ cells per transwell insert and cultured for 48 h in KSFM. Once confluent, cells were differentiated in calcium‐containing DMEM to promote barrier formation. Cells were treated with various concentrations of IL‐1β (10, 100 or 1000 pg/mL) in differentiation media at 48 h post‐differentiation. TEER was measured daily at the same time using an EVOM2 volt/ohmmeter (World Precision Instruments) for a total of 6 days. The media was refreshed every 2 days.

### 2.6 General Protease Activity Assay

Supernatant (500 μL) was harvested from untreated and treated KC cultures. The medium was centrifuged at 10,000 g for 5 min at room temperature (RT) to remove cell debris. Following centrifugation, 350 μL of clarified supernatant was collected for analysis. General proteolytic activity was measured using the Protease Activity Assay Kit (Cat # ab112152, Abcam) following the manufacturer's instructions. Briefly, 50 μL of KC ± treatment supernatant was added to a 96‐well plate and mixed with an equal volume of 1:100 diluted fluorescent casein substrate in 2X reaction buffer solution. The plate was incubated at RT for 48 h, protected from light. Fluorescence was measured at 24 and 48 h post incubation on a SpectraMax i3x plate reader (Molecular Devices) with excitation and emission wavelengths of 490 and 525 nm, respectively. Relative fluorescence units (RFU) from wells containing cell‐free culture medium with substrate were used as background controls and subtracted from all sample readings before analysis. For yeast‐exposed samples, RFU from yeast‐only wells was also subtracted. A 1 μL volume of trypsin (provided in the assay kit at 5 U/μL) was included as a positive control for all assays.

### 2.7 Total Protein Isolation and Western Blot Analysis

KC protein lysates were prepared using 100 μL radioimmunoprecipitation (RIPA) buffer (Cat # BP‐115D, Boston BioProducts) supplemented with 1 μL phosphate inhibitor cocktail (Cat # P5726, Sigma‐Aldrich), 1 μL protease inhibitors cocktail (Cat # P8340, Sigma‐Aldrich) and 0.2% SDS. RIPA buffer mixture was added to each well and KC were lysed by incubating at 4°C for 15 min with gentle rocking every 5 min. Lysates were collected by pipetting up and down five times and transferred to microcentrifuge tubes. Cell debris was removed by centrifugation at 13,000 g for 5 min at 4°C. The collected lysates were stored at −80°C. For western blotting, 15 μL of cell lysate was mixed with 5 μL 4X SDS bolt dye (Cat # B0007, Invitrogen) and 2 μL 10X DTT reducing agent (Cat # B0009, Invitrogen). Samples were heated for 5 min at 95°C before loading into NuPAGE 4%–12% Bis‐Tris gels (Cat # NW04120BOX, Invitrogen). Electrophoresis was performed at 160 V over an 8 cm gel for 40 min, followed by transfer onto a polyvinylidene difluoride (PVDF) membrane (Cat # 88520, Thermo Fisher Scientific). Membranes were blocked with 5% dry milk in PBS for 1 h at RT and then incubated overnight at 4°C with primary antibodies at the specified dilutions (Table S2). Following primary antibody incubation, membranes were washed three times for 10 min each with PBS containing 0.05% Tween 20. Subsequently, secondary antibodies were applied for 1 h at RT or overnight at 4°C, followed by three additional washes. Protein size was determined with the See Blue Plus 2 ladder (Cat # LC5925, Invitrogen) and bands were visualised using SuperSignal West Pico PLUS Chemiluminescent Substrate (Cat # 34577, ThermoFisher Scientific). Colorimetric and chemiluminescent images were captured using the BioRad Gel Doc XR Imaging System. Protein expression was normalised to β‐actin, and relative protein levels were quantified by densitometry using ImageJ software.

### 2.8 IL‐1β and CXCL8 Quantitation

IL‐1β and CXCL8 protein levels in KC‐yeast culture supernatants were measured using two ELISA kits (IL‐1β: Cat # DY201‐05, R&D Systems; CXCL8: Cat # 431501, BioLegend, respectively) following the manufacturer's protocol. Briefly, 96‐well microplates were coated overnight at RT with capture antibody (100 μL/well in PBS). Plates were then washed three times with PBS containing 0.05% Tween 20 and blocked with 150 μL/well of 1% BSA in PBS (0.2 μm filtered) for 1 h. Subsequently, 100 μL/well of samples or cytokine standards (IL‐1β: 3.9–250 pg/mL; CXCL8: 15.16–1000 pg/mL) were added and incubated for 2 h at RT on a microplate vortex mixer (Cat # 89399–882, VWR International). After five washes, plates were incubated with 100 μL/well of detection antibody diluted in blocking buffer for 2 h at RT. Following another five washes, 100 μL/well of Streptavidin‐HRP (1:40 dilution in blocking reagent) was added and incubated for 20 min under light‐protected conditions. Plates were then washed four times, and 50 μL/well of TMB substrate was added to each well. The reaction was stopped 15 min post incubation by adding 50 μL of 2 N H_2_SO_4_. Absorbance was measured at 450 nm using a Spectramax i3X and cytokine concentration was calculated using the standard curves.

### 2.9 RT‐qPCR

KC exposed to either yeast or cytokines for 24 h were washed with 500 μL of PBS prior to the addition of 250 μL TRI Reagent Solution (Cat # 93289, Millipore Sigma). Cells were mechanically scraped using a 1 mL syringe plunger, and lysates were transferred to a microcentrifuge tube. Total mRNA was extracted using an mRNA isolation kit (Cat # R683402, Omega Biotek) following the manufacturer's protocol, then eluted in 35 μL of nuclease‐free water and quantified using a NanoDrop Lite (ThermoFisher Scientific). Complementary DNA (cDNA) was synthesised from 300 ng of mRNA using the qScript cDNA synthesis kit (Cat # 95047–500, Quantabio) in a total volume of 20 μL. The reaction conditions were as follows: 22°C for 5 min, 42°C for 30 min, and 85°C for 5 min. For quantitative PCR (qPCR) analysis, reaction mixtures were prepared in 96‐well plates. Each well contained 4 μL of diluted cDNA (0.4 μL cDNA in 3.6 μL nuclease‐free water) and 6 μL of primer mix consisting of 5 μL PerfeCTa SYBR Green SuperMix (Cat # 95053‐02 K, Quantabio) and 1 μL gene‐specific primers (10 μM) as listed in Table S3. RT‐qPCR was performed on a CFX Connect Real‐Time PCR Detection System (Cat # 1855201, Bio‐Rad) using the following protocol: 94°C for 3 min, 39 amplification cycles (94°C for 15 s and 55°C for 1 min), 95°C for 1 min and 55°C for 1 min. Gene expression was normalised to *HPRT1* and reported as 2^−∆∆Cq^.

### 2.10 Data and Statistical Analyses

**FIGURE 1:**
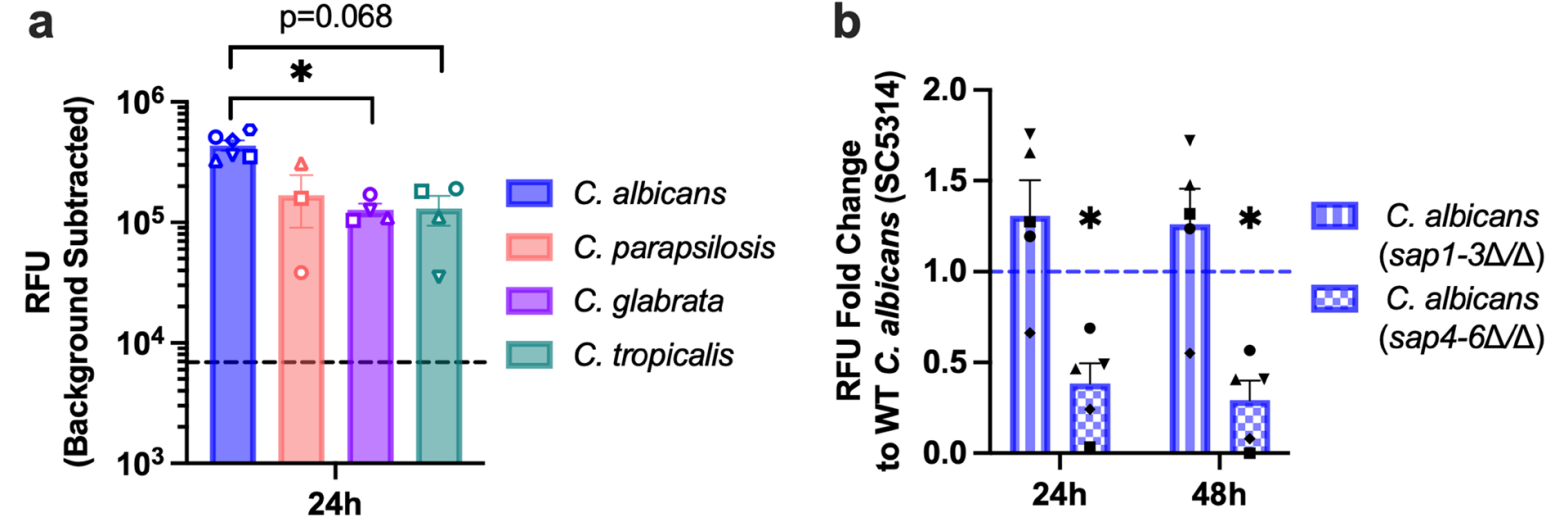
Enhanced protease activity in KC‐ *C. albicans* co‐cultures is predominantly induced by fungal Sap4–6. Protease activity from N/TERT‐2G keratinocytes (KC) was measured after 24 h of yeast exposure (10^3^ colony forming units [CFU]) and displayed as relative fluorescence units (RFU). (a) KC were co‐cultured with different *Candida* species. Each symbol represents an individual strain within a species, and data points show the mean from two independent experiments. The dashed line represents the amount of protease activity detected in non‐yeast exposed controls. (b) Fold change in protease activity between wild‐type (SC5314) and recombinant strains containing *sap1‐3* or *sap4‐6* triple deletions (*n =* 5 experiments). *p < 0.05. *sap*, Secreted aspartyl proteinase.

**FIGURE 2:**
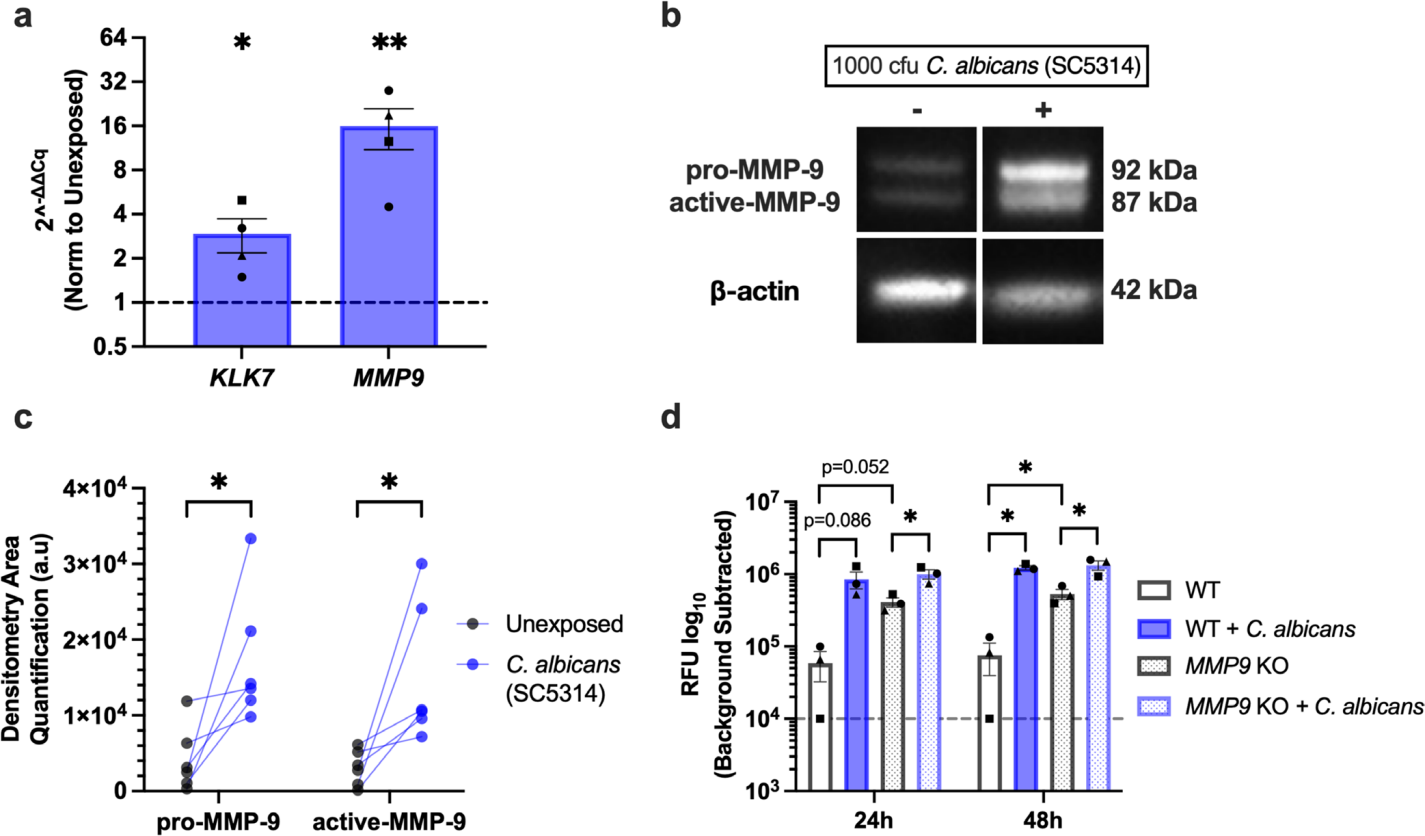
*C. albicans* ‐induced MMP‐9 expression amplifies KC proteolytic response. (a) Changes in KC expression of *KLK7* and *MMP9* mRNA following 24 h exposure to *C. albicans* (*n* = 4 experiments). (b) Representative immunoblot of pro‐ and active MMP‐9 in KC after *C. albicans* exposure. (c) Quantification of MMP‐9 expression via densitometry with β‐Actin as a loading control (*n* = 6 experiments). (d) Measurement of protease activity in wild‐type (WT) and *MMP9* knockout (KO) KC cocultured with *C. albicans* (*n =* 3 experiments). The dashed line represents an arbitrary amount of protease activity given to samples with values below the limit of detection. *p < 0.05, **p < 0.01. *KLK7*, Kallikrein‐related peptidase 7, *MMP9*, Matrix metalloproteinase‐9.

**FIGURE 3:**
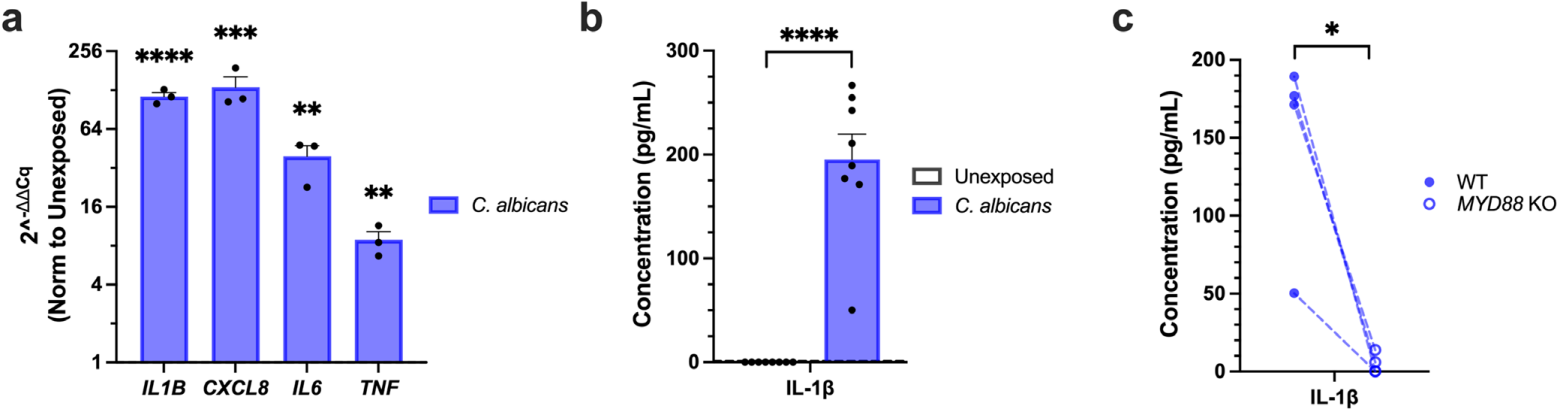
*C. albicans* promotes release of IL‐1β via a MyD88‐dependent pathway in KC. (a) mRNA expression of *IL1B*, *CXCL8*, *IL6*, and *TNF* in KC following 24 h of *C. albicans* exposure (*n* = 3 experiments). (b) IL‐1β protein secretion in KC–yeast co‐cultures (*n* = 8 experiments). (c) Quantification of secreted IL‐1β in supernatants of WT and *MYD88* KO KC post *C. albicans* colonization (*n* = 4 experiments). *p < 0.05, **p < 0.01, ***p < 0.001, ****p < 0.0001.

**FIGURE 4:**
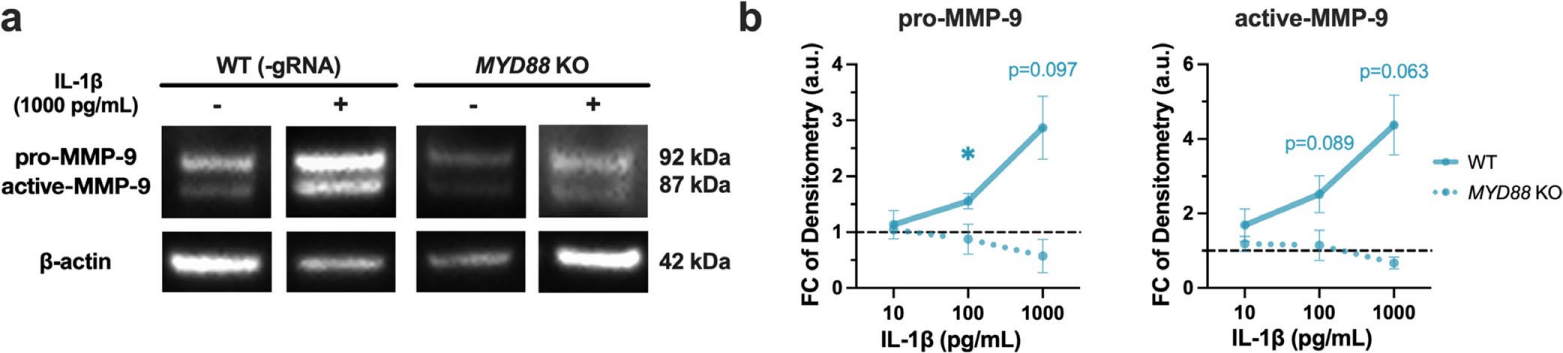
MyD88 signalling drives MMP‐9 production in IL‐1β‐stimulated KC. KC were treated with recombinant IL‐1β (10–1000 pg/mL) for 24 h. (a) Representative immunoblot of pro‐ and active MMP‐9 after cytokine treatment. (b) Quantification of MMP‐9 expression by densitometry is displayed as fold change relative to untreated controls (*n* = 4 experiments). *p < 0.05.

**FIGURE 5:**
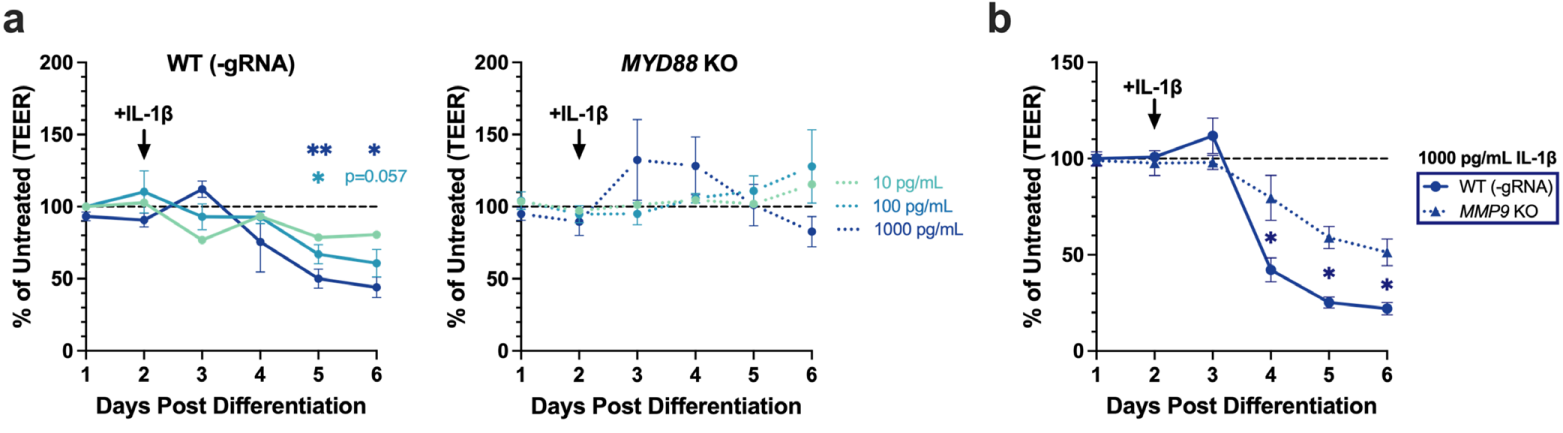
MMP‐9 contributes to IL‐1β–mediated barrier dysfunction downstream of MyD88. WT and *MYD88* KO KC cultures were differentiated for 6 days and assessed for barrier function via transepithelial electrical resistance (TEER). (a) Recombinant IL‐1β (10–1000 pg/mL) treatment was introduced at day 2 post differentiation (10 pg/mL: *N* = 1 experiment, 100–1000 pg/mL: *N* = 3 experiments). (b) TEER in WT and *MMP9* KO KC treated with 1000 pg/mL of IL‐1β (*n* = 3 experiments). TEER values were normalized to untreated controls (dashed line). *p < 0.05, **p < 0.01.

All statistical tests were conducted with GraphPad Prism software v10.4.1 (GraphPad). Western blot (arbitrary units from densitometry), protease activity (relative fluorescent units), and ELISA (pg/mL) statistical analyses were performed on raw values. RT‐qPCR data were analysed using ∆Cq values (Cq of gene of interest minus Cq of housekeeping gene [*HPRT1*]) between unexposed and *C. albicans* colonized conditions. Display of qPCR data was done using the 2^−∆∆Cq^ format, with mRNA levels from *Candida*‐colonized samples expressed relative to unexposed levels (fold change). TEER values were reported in ohms·cm^2^. For knockout (KO) KC experiments, data were normalised to wildtype (WT). In Figure 1b, fold change values were calculated relative to the parental strain *C. albicans* SC5314. Group comparisons were conducted using the Kruskal–Wallis test (Figure 1a), two‐way ANOVA (Figures 1b, 4 and 5a; Figure S7), or paired *t*‐tests (Figures 2, 3 and 5b; Figures S1 and S6), as appropriate. A *p* < 0.05 was considered statistically significant. Outliers were identified using ROUT analysis with a Q‐value of 1% and subsequently excluded from further analysis in Figures 3b,c and 4b [36]. Data are presented as mean ± SEM for all figures with *n* ≥ 3, and as mean values for figures with *n* < 3. Each individual data point represents the average value from one biological experiment consisting of two to three experimental replicates.

### 2.11 Ethics Statement

The authors confirm that the ethical policies of the journal, as noted on the journal's author guidelines page, have been adhered to. No ethical approval was required as the research in this article related to microorganisms.

## 3 Results

### 3.1 Enhanced Proteolytic Activity in KC‐ *C. albicans* Co‐Cultures Relies on Expression of Host MMP‐9 and Fungal Sap4‐6

To understand whether various skin resident *Candida* species alter epidermal protease activity, we collected supernatants from KC exposed to *C. albicans* , *C. tropicalis* , *C. parapsilosis,* or *C. glabrata* . Skin cells exposed to *C. albicans* demonstrated a significant increase in proteolytic activity compared to unexposed controls (Figure S1). To ensure that this effect was not strain specific, we compared the commonly used virulent reference strain SC5314 of *C. albicans* with four clinical isolates (one from the URMC microbiology clinical unit and three from the University of Exeter). Our results indicated that all strains increased KC protease activity, and such enhancement was more robust than that induced by other *Candida* species tested (Figure 1a).

We next investigated whether the expression of specific *C. albicans* virulence‐associated genes mediated the observed elevation in KC proteolytic activity, with a focus on *sap1‐6*. Different Saps are produced at various stages of *C. albicans* tissue invasion, with Sap1‐3 linked to expression in yeast cells and with adhesion, and Sap4‐6 associated with hypha formation. Using *sap*‐deleted strains targeting these clusters [29, 37], we observed that the *sap4‐6*∆/∆ null strain induced significantly lower KC protease activity compared to WT (Figure 1b). We also tested a candidalysin‐deficient strain (*ece1*∆/∆), as this virulence factor is critical in *C. albicans* pathogenicity [38]. In two separate experiments, we observed a similar increase in proteolytic activity from cultures exposed to the mutant strain (Figure S2). These findings suggest that *C. albicans* is a potent inducer of KC protease activity and that Saps4, 5, and/or 6 are the primary drivers of this host cell response to colonization.

To identify individual host enzymes responsible for *C. albicans‐*induced KC proteolytic activity, we assessed the transcription of KLK7, MMP‐9, and TIMP‐1 (a protease inhibitor that regulates MMP activity) because of their known association with AD. Exposure of KC to *C. albicans* significantly increased *KLK7* and *MMP9* expression (Figure 2a), while *TIMP1* remained unchanged (not shown). We then focused on MMP‐9 protein for subsequent analyses because its transcript was substantially induced by *C. albicans* . Given that most proteases are synthesized as inactive precursors requiring further cleavage to be functionally active [39], we examined the expression of both the pro‐ and active forms of MMP‐9 via western blot. Both forms were increased in KC–C. *albicans* co‐cultures, indicating that not only was production enhanced, but processing to a functional enzymatic state was also occurring (Figure 2b,c).

Since MMP‐9 was elevated at both the gene and protein levels, we hypothesized that it contributed to *C. albicans* ‐induced KC proteolytic activity. To test this, we generated *MMP9* knockout (KO) KC using CRISPR/Cas9 according to our established methodology [34], and validated gene deletion by PCR and western blot (Figure S3a,b). While the magnitude of protease activity (measured in raw RFU) following *C. albicans* exposure remained unchanged between WT and *MMP9* KO KC, we observed that *MMP9* KO KC displayed significantly higher baseline RFU. Consequently, the fold change in protease activity induction following *C. albicans* exposure was substantially reduced upon loss of *MMP9* (Figure 2d). These findings suggest that MMP‐9 expression amplifies KC protease activity following *C. albicans* exposure.

### 3.2 *C. albicans* Initiates Release of IL‐1β via a MyD88‐Dependent Pathway in KC

Abnormal protease levels in cutaneous diseases such as bullous pemphigoid and AD have been positively correlated with local pro‐inflammatory cytokine expression [26, 40]. Hence, we screened changes in KC mRNA of the prototypical pro‐inflammatory cytokines and chemokines (*CXCL8, IL6, IL1B, and TNF*) in response to *C. albicans* . All tested inflammation‐related genes were significantly upregulated, with *IL1B and CXCL8* showing the greatest increases (Figure 3a). In the context of AD, *IL1B* expression has been reported in lesional skin [40, 41], and serum IL‐1β levels positively correlate with disease severity [42]. We next asked whether the observed increase in *IL1B* transcription also resulted in greater cytokine secretion. Since pro‐IL‐1β requires cleavage to become biologically active [43], we selected a mature IL‐1β‐specific ELISA for quantification. Using this assay, we detected enhanced secretion of IL‐1β in KC supernatants following *C. albicans* exposure (Figure 3b). Additionally, we confirmed elevated levels of CXCL8 in KC‐ *C. albicans* co‐culture supernatants (Figure S4).

To uncover the molecular pathway responsible for increased IL‐1β production, we focused on the adaptor protein MyD88, which mediates signalling through both toll‐like receptors (TLRs) and IL‐1R [44, 45]. We generated a *MYD88* KO cell line and validated gene disruption by PCR (Figure S3a). After exposing these cells to *C. albicans* , we observed a pronounced reduction in IL‐1β transcription (Figure S5), and secretion (Figure 3c), compared to WT controls. While previous studies implicated MMP‐9 activity as an alternative route for IL‐1β processing [46], IL‐1β production following *C. albicans* colonization was unaffected in *MMP9* KO cells (Figure S6). These results indicate that MyD88 signalling is essential for elevated IL‐1β secretion from KC in response to *C. albicans* .

### 3.3 MMP‐9 Drives Barrier Dysfunction Through KC IL‐1β/MyD88 Signalling

Expression of pro‐inflammatory cytokines, including IL‐1β, is known to modulate host proteolytic activity in various cell types [47, 48]. Given the substantial induction of IL‐1β mRNA and protein following *C. albicans* exposure, we hypothesized that IL‐1β contributes to KC protease activation. To test this, we treated WT and *MYD88* KO KC with varying doses of recombinant IL‐1β (10–1000 pg/mL) for 24 h. At concentrations consistent with those induced by *C. albicans* (~200 pg/mL), IL‐1β treatment increased both pro–MMP‐9 and active MMP‐9 in WT KC (pro–MMP‐9: 1.56 ± 0.14‐fold at 100 pg/mL and 2.87 ± 0.56‐fold at 1000 pg/mL; active MMP‐9: 2.52 ± 0.49‐fold and 4.37 ± 0.80‐fold, respectively; mean ± SEM; Figure 4a,b). Furthermore, this induction was absent in cells lacking MyD88 expression (Figure 4b). These observations support the importance of IL‐1R/MyD88 signalling in regulating MMP‐9 expression in KC.

Elevated protease activity and inflammatory cytokine levels are associated with barrier dysfunction in AD patients [27, 49]. To ascertain IL‐1β's impact on epidermal barrier function, we evaluated transepithelial electrical resistance (TEER), a sensitive in vitro measurement of epithelial barrier function [35, 50], over 6 days of KC differentiation with or without IL‐1β treatment. The addition of IL‐1β reduced TEER at multiple timepoints post differentiation (Figure 5a). We hypothesized this decrease reflected altered KC differentiation, as evidenced by decreased expression of cytokeratin‐10 (Figures S5 and S7), a marker associated with KC transitioning from a proliferative to a differentiating state [51]. Conversely, *MYD88* KO KC treated with IL‐1β maintained their barrier function and cytokeratin‐10 expression at levels comparable to untreated controls (Figures S5 and S7). Since we observed the impairment of barrier function through IL‐1β/MyD88 signalling, and we had also observed this pathway to regulate MMP‐9 expression (Figure 4a,b), we further investigated whether MMP‐9 activity contributed to KC barrier disruption. Treatment of WT and *MMP9* KO KC with IL‐1β revealed partial restoration of barrier function in *MMP9* KO KC (Figure 5b). Collectively, our data suggest that MyD88 plays a previously unappreciated role in epidermal differentiation during inflammation and that MMP‐9 directly contributes to KC barrier impairment.

## 4 Discussion and Conclusions

**FIGURE 6:**
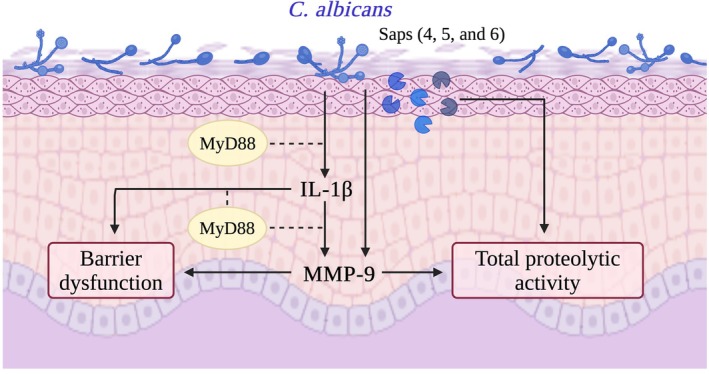
Proposed pathway through which *C. albicans* colonization leads to epidermal barrier disruption and proteolytic activation. During *C. albicans* colonization, fungal Sap4–6 expression enhances KC proteolytic activity, which is further amplified through host MMP‐9 activation. This synergistic cascade disrupts skin homeostasis, exemplified by MyD88‐dependent IL‐1β release, greater MMP‐9 induction, and ultimately impaired barrier function. Together, these findings illustrate how aberrant *C. albicans* colonisation may promote AD‐like in vitro pathology.

Understanding how *C. albicans* influences KC proteolytic activity and inflammation provides new insights into cutaneous diseases with an altered mycobiome. Here, we demonstrated for the first time that *C. albicans* enhances KC proteolytic activity, with Sap 4, 5 and/or 6 serving as key mediators. This response is further amplified by host MMP‐9 expression, which is also induced upon *C. albicans* exposure. Mechanistically, *C. albicans* promotes IL‐1β secretion from KC through MyD88 signalling, likely acting in an auto/paracrine manner to further enhance MMP‐9 expression. Finally, MMP‐9 contributes to IL‐1β–mediated barrier dysfunction. A summary of this pathway is shown in Figure 6. Since epidermal inflammation and heightened proteolytic activity are two characteristics of AD, our findings advance the current understanding of how cutaneous microorganisms, that is, *C. albicans* , could contribute directly to AD pathogenesis.

A key finding of our study is that *C. albicans* strains producing Sap4‐6 significantly enhance KC protease activity. Of note, *sap4‐6* expression varies among strains and can be absent in clinical commensal isolates [52, 53]. This expression variability likely explains why a varying amount of protease activity was detected across KC‐clinical isolate co‐cultures (Figure 1a). Supporting the role of Sap4‐6 in modulating host protease activity and inflammation, Sap6 has been shown to degrade cystatin A, a skin cysteine protease inhibitor [54], and activate protease‐activated receptor 2 in oral epithelial cells, driving excessive CXCL8 release [20]. While these studies examined individual Sap activity on host cell responses using recombinant enzymes, they did not fully capture the dynamic host–microbe interactions during colonization. In contrast, our results provide novel insight into how host proteolytic responses change following live *Candida* exposure. Interestingly, we noticed that mutant strains deficient in Sap1‐3 triggered a greater KC proteolytic response than the WT SC5314 strain in our experiments. This could be due to compensatory upregulation of *sap4*, *sap5*, and/or *sap6* in response to the loss of *sap1‐3*, which has been supported by increased *sap5* expression observed in *sap1/3∆/∆* double KO mutants [55]. Importantly, the SC5314 strain of *C. albicans* has been found to be RNAi‐deficient, which may lead to greater transcription of Saps and therefore proteolytic activity compared to RNAi‐active clinical isolates [56]. Nevertheless, the additional clinical isolates we investigated demonstrated a consistent stimulation of host protease activity, suggesting that RNAi status likely does not confound the association between *sap4‐6* and KC proteolytic activity. Future studies can further validate our observations by examining a broader range of clinical isolates or by performing *sap4‐6* transfection experiments. Additionally, conducting the same analyses using single *sap* deletion and/or overexpression mutant strains would further clarify the contributions of specific *sap* genes to KC protease activation. Translationally, qPCR measurements of *C. albicans* isolates from AD skin could uncover the *sap4‐6* expression patterns in a disease‐relevant context.

By monitoring changes in host‐derived proteases and their inhibitors that could explain the observed enhancement in total protease activity, we found that KC exposed to *C. albicans* exhibited a notable increase in MMP‐9. This protease is a key modulator of pro‐inflammatory cytokine processing and tissue remodelling and has often been implicated in cutaneous diseases [24, 57]. Using an MMP‐specific enzymatic assay, one study demonstrated that AD lesions exhibit 10‐ to 24‐fold higher MMP activity than healthy controls, with MMP‐8 and MMP‐9 contributing the most [25]. This suggests that MMP‐9 could serve as a potential biomarker for AD pathology. Unexpectedly, depletion of MMP‐9 in KC resulted in an increase in baseline proteolytic activity. A possible explanation for this phenomenon could be functional compensation by other skin proteases. MMP‐2, a closely related gelatinase, can compensate for MMP‐9 in extracellular matrix remodelling, which has been demonstrated in murine KC [58]. Similarly, studies in pancreatic islets have reported that tumors lacking MMP‐9 display increased cathepsin B activity [59]. Despite the baseline activity alteration in *MMP9* KO KC, these cells showed a reduced fold‐change in proteolytic response to *C. albicans* . This suggests that MMP‐9 is integral to the responsiveness to yeast exposure. Collectively, our findings indicate that MMP‐9 amplifies *C. albicans* ‐triggered proteolytic activity in KC, which is important since this protein is crucial for tissue remodelling, inflammatory modulation, and skin barrier function [60].

While both IL‐1β and CXCL8 showed pronounced transcriptional induction among the pro‐inflammatory cytokines we analyzed, we prioritized IL‐1β for further investigation due to its distinct and mechanistically relevant roles in AD pathogenesis. Specifically, IL‐1β serves as a first‐line inflammatory mediator in acute AD by upregulating TSLP [61], an alarmin that promotes type 2 inflammation. It also promotes IL‐23 production [62], supporting Th22 responses that are increasingly implicated in AD severity [63, 64] Additionally, elevated expression of IL‐1β has been detected in skin biopsies and serum from patients with Netherton syndrome and AD [41, 65], and blocking IL‐1 signalling with anakinra (a human IL‐1 receptor antagonist) alleviates disease severity in patients with Netherton syndrome [66]. Given the growing recognition of IL‐1β in cutaneous diseases, our data suggest that *C. albicans* may exacerbate disease pathology by inducing its secretion, similar to that of *Malassezia* spp., which have long been implicated in head and neck AD [ 8, 67, 68]. *C. albicans* expresses multiple pathogen‐associated molecular patterns that stimulate IL‐1β production in immune cells via TLR/MyD88 [44, 69], or C‐type lectin receptor (CLR) signalling [70, 71]. While these pathways have been well characterized in cells of hematopoietic origin, their role in KC and skin inflammation remains underexplored. Here, we show that IL‐1β induction in KC by *C. albicans* is MyD88‐dependent, resembling the response observed in hematopoietic cells. Notably, pathogen‐associated molecular patterns associated with MyD88 signalling have been proposed to prime *IL1B* gene expression and pro‐IL‐1β synthesis, while the processing and release of mature IL‐1β are often facilitated by damage‐associated molecular pattern‐dependent inflammasome activation through Nod‐like receptors (NLRs), particularly NLRP3 [72]. Studies in phagocytic cells suggest that the second signal is dependent on the hyphal form of *C. albicans* [ 69, 70]. More contemporary findings further expand on this, demonstrating that activation is specifically mediated by the fungal toxin candidalysin, which is released from hyphae [73, 74]. Therefore, future studies should investigate whether *C. albicans* colonization is associated with greater NLRP3 inflammasome activation in the human epidermis. Consistent with our findings, a study co‐culturing *Malassezia* spp. with HaCat KC also reported elevated IL‐1β [68]. Together, these findings suggest that IL‐1β modulation could be a shared mechanism by which commensal fungal species exacerbate AD pathology.

In congruence with this finding, our results demonstrated that IL‐1β/MyD88 signalling compromises KC barrier function, with MMP‐9 serving as a downstream effector. While inhibition of IL‐1R/MyD88 signalling has been shown to restore diminished barrier protein expression in AD models [75, 76], our findings suggest MMP‐9 could serve as an alternative target for mitigating barrier impairment in inflamed skin. In line with our diminished TEER observations, a study on Crohn's disease indicated that MMP‐9 produced by Caco‐2 epithelial cells disrupts tight junctions and reduces barrier function [77]. Therefore, future investigations on the direct impact of MMP‐9 on KC barrier proteins could further elucidate its role in barrier regulation.

In summary, our study emphasizes the underappreciated role that a fungal skin commensal plays in inducing epidermal inflammation and ultimately dysregulating barrier homeostasis. By elucidating the interaction between fungi and host innate immune responses, we propose the need for greater inclusion of mycobiome‐focused skin research and targeted therapeutic interventions in the future management of inflammatory skin diseases.

## Author Contributions

**Jingyi Wang:** conceptualization, investigation, writing – original draft, methodology, visualization, writing – review and editing, formal analysis, data curation. **Neil A. R. Gow:** methodology, resources, writing – review and editing. **Matthew G. Brewer:** investigation, funding acquisition, writing – original draft, methodology, writing – review and editing, formal analysis, project administration, data curation, resources, supervision.

## Funding

This work was supported by Wellcome Trust, National Institute for Health and Care Research, and Medical Research Council Centre for Medical Mycology.

## Conflicts of Interest

The authors declare no conflicts of interest.

## Supporting information


**Figure S1:** Enhanced protease activity is detected in KC‐ *C. albicans* co‐cultures. Protease activity from N/TERT‐2G keratinocytes (KC) was measured after 24 h of exposure to the *C. albicans* strain SC5314 (10^3^ colony forming units [CFU]) and displayed as relative fluorescence units (RFU). *n* = 3 experiments.
**Figure S2:** Candidalysin expression by *C. albicans* is dispensable for inducing KC protease activity. Protease activity from N/TERT‐2G keratinocytes (KC) was measured after 24 h of exposure (10^3^ colony forming units [CFU]) to either wildtype (SC5314/CAI4) or *ece1*Δ/Δ null (candidalysin lacking) *C. albicans* and displayed as relative fluorescence units (RFU). *n* = 2 experiments.
**Figure S3:** Confirmation of CRISPR/Cas9‐Based KO of *MMP9* and *MYD88* in N/TERT‐2G KC. (a) PCR amplification and gel electrophoresis were performed to verify the KO of *MMP9* and *MYD88* in KC. Lane 1: Ladder; Lane 2–3: WT sample (without guide RNA [‐gRNA]) and polyclonal (p) *MMP9* KO KC were evaluated for *MMP9* and Lane 4–5: WT (‐gRNA) and p*MYD88* KO KC evaluated for *MYD88* gene editing. (b) Western blot analysis was performed to determine MMP‐9 protein expression in *MMP9* KO KC compared to WT; β‐actin was used as a loading control.
**Figure S4:** *C. albicans* promotes release of CXCL8 from KC. CXCL8 protein secretion following 24 h of KC–yeast co‐culture (*n* = 3 experiments).
**Figure S5:** *MYD88*‐Mediated regulation of *IL1B*, *MMP9* and *KRT10* transcription in KC. RT‐qPCR was performed to assess the relative expression of *IL1B*, *MMP9* and *KRT10* in WT (‐gRNA) and *MYD88* KO KC after 24 h of co‐culture with *C. albicans* . Expression levels for genes of interest were normalized to the housekeeping gene *HPRT1* (*MMP9* & *KRT10*: *n* = 2 experiments; *IL1B*: *n* = 3 experiments).
**Figure S6:** Loss of MMP‐9 does not affect *C. albicans* ‐induced IL‐1β secretion. Quantification of secreted IL‐1β in supernatants of WT and *MMP9* KO KC following 24 h of *C. albicans* colonization (*n* = 3 experiments).
**Figure S7:** IL‐1β treatment diminishes cytokeratin‐10 expression through MyD88 signalling. (a) Representative immunoblot showing cytokeratin‐10 expression in WT and *MYD88* KO KC treated with IL‐1β (10–1000 pg/mL) for 24 h. (b) Quantification of cytokeratin‐10 expression relative to untreated controls using densitometry (10 & 1000 pg/mL: *n* = 3 experiments; 100 pg/mL: *n* = 4 experiments). *p < 0.05.
**Table S1:** Candida strains used in this study.
**Table S2:** Antibodies used for western blot.
**Table S3:** Primers used for RT‐qPCR.

## Data Availability

The data that support the findings of this study are available from the corresponding author upon reasonable request.
