# Supplementary material for: Candida albicans Enhances Protease Activity and Activates MyD88‐Dependent IL‐1β Production in Human Keratinocytes
Source: Mycoses. 2025 Nov 26;68(11):e70133. doi: 10.1111/myc.70133 (PMC12650128; doi:10.1111/myc.70133)
Supplement: Supplementary file 1 — Figure S1: Enhanced protease activity is detected in KC‐ C. albicans co‐cultures. Protease activity from N/TERT‐2G keratinocytes (KC) was measured after 24 h of exposure to the C. albicans strain SC5314 (103 colony forming units [CFU]) and displayed as relative fluorescence units (RFU). n = 3 experiments. Figure S2: Candidalysin expression by C. albicans is dispensable for inducing KC protease activity. Protease activity from N/TERT‐2G keratinocytes (KC) was measured after 24 h of exposure (103 colony forming units [CFU]) to either wildtype (SC5314/CAI4) or ece1Δ/Δ null (candidalysin lacking) C. albicans and displayed as relative fluorescence units (RFU). n = 2 experiments. Figure S3: Confirmation of CRISPR/Cas9‐Based KO of MMP9 and MYD88 in N/TERT‐2G KC. (a) PCR amplification and gel electrophoresis were performed to verify the KO of MMP9 and MYD88 in KC. Lane 1: Ladder; Lane 2–3: WT sample (without guide RNA [‐gRNA]) and polyclonal (p) MMP9 KO KC were evaluated for MMP9 and Lane 4–5: WT (‐gRNA) and pMYD88 KO KC evaluated for MYD88 gene editing. (b) Western blot analysis was performed to determine MMP‐9 protein expression in MMP9 KO KC compared to WT; β‐actin was used as a loading control. Figure S4: C. albicans promotes release of CXCL8 from KC. CXCL8 protein secretion following 24 h of KC–yeast co‐culture (n = 3 experiments). Figure S5: MYD88‐Mediated regulation of IL1B, MMP9 and KRT10 transcription in KC. RT‐qPCR was performed to assess the relative expression of IL1B, MMP9 and KRT10 in WT (‐gRNA) and MYD88 KO KC after 24 h of co‐culture with C. albicans . Expression levels for genes of interest were normalized to the housekeeping gene HPRT1 (MMP9 & KRT10: n = 2 experiments; IL1B: n = 3 experiments). Figure S6: Loss of MMP‐9 does not affect C. albicans ‐induced IL‐1β secretion. Quantification of secreted IL‐1β in supernatants of WT and MMP9 KO KC following 24 h of C. albicans colonization (n = 3 experiments). Figure S7: IL‐1β treatment diminishes cytokeratin‐10 [file MYC-68-e70133-s001.docx]

**Supplementary Information**

**
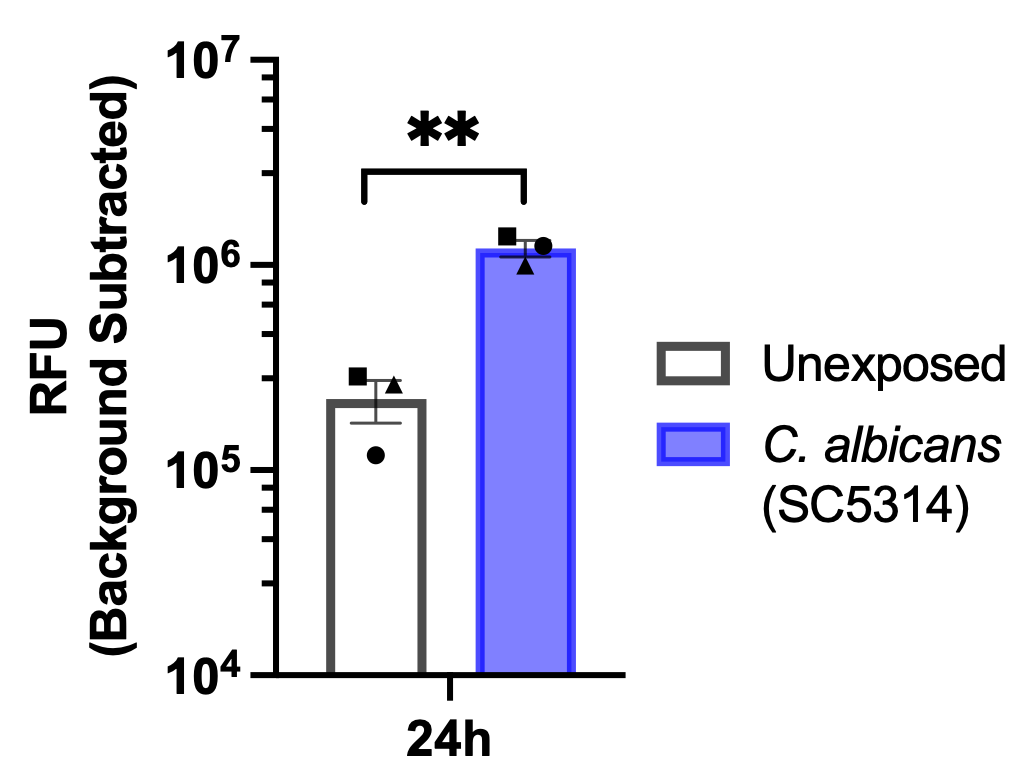
**

**Supplementary Figure 1. Enhanced protease activity is detected in KC-*C. albicans* co-cultures.** Protease activity from N/TERT-2G keratinocytes (KC) was measured after 24 hours of exposure to the *C. albicans* strain SC5314 (10³ colony forming units [CFU]) and displayed as relative fluorescence units (RFU). *n*=3 experiments.


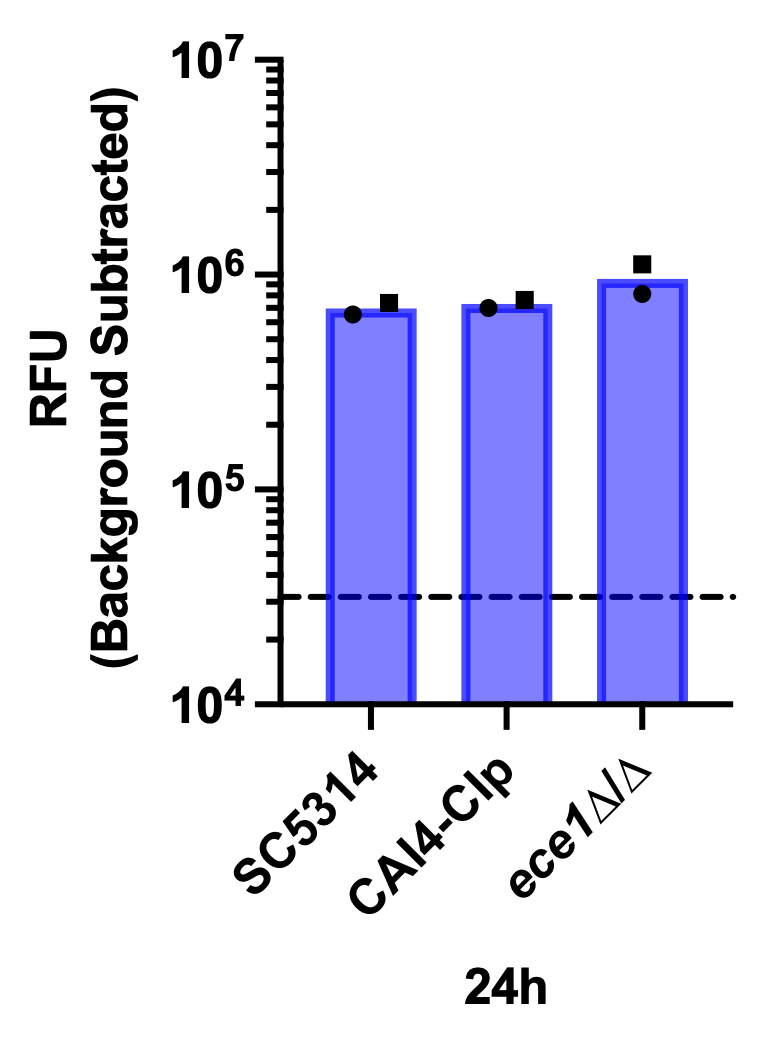


**Supplementary Figure 2. Candidalysin expression by *C. albicans* is dispensable for inducing KC protease activity.** Protease activity from N/TERT-2G keratinocytes (KC) was measured after 24 hours of exposure (10³ colony forming units [CFU]) to either wildtype (SC5314/CAI4) or *ece1*Δ/Δ null (candidalysin lacking) *C. albicans* and displayed as relative fluorescence units (RFU). *n*=2 experiments.


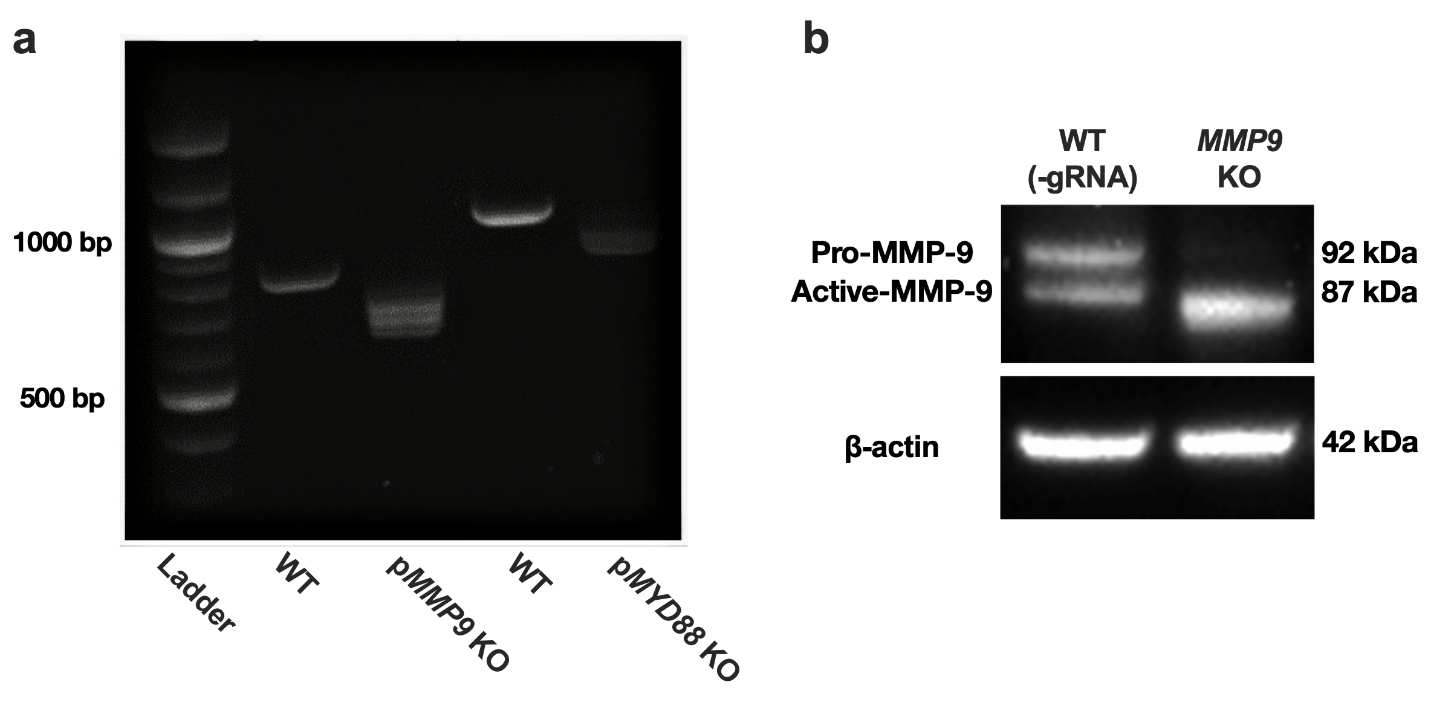


**Supplementary Figure 3. Confirmation of CRISPR/Cas9-Based KO of *MMP9* and *MYD88* in N/TERT-2G KC.** (a) PCR amplification and gel electrophoresis were performed to verify the KO of *MMP9* and *MYD88* in KC. Lane 1: Ladder; Lane 2-3: WT sample (without guide RNA [-gRNA]) and polyclonal (p) *MMP9* KO KC were evaluated for *MMP9* and Lane 4-5: WT (-gRNA) and p*MYD88* KO KC evaluated for *MYD88* gene editing. (b) Western blot analysis was performed to determine MMP-9 protein expression in *MMP9* KO KC compared to WT; β-actin was used as a loading control.


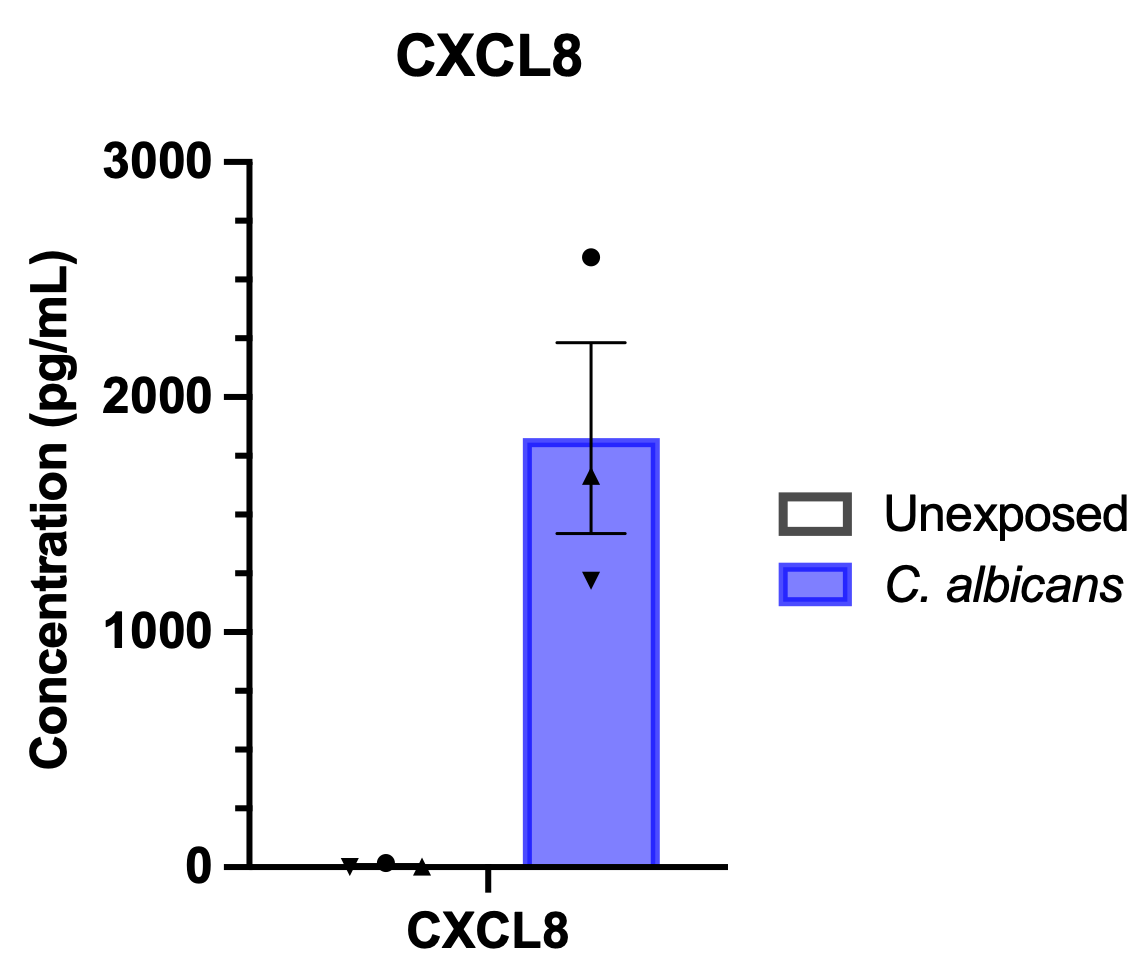


**Supplementary Figure 4. *C. albicans* promotes release of CXCL8 from KC.** CXCL8 protein secretion following 24 hours of KC–yeast co-culture (*n*=3 experiments).


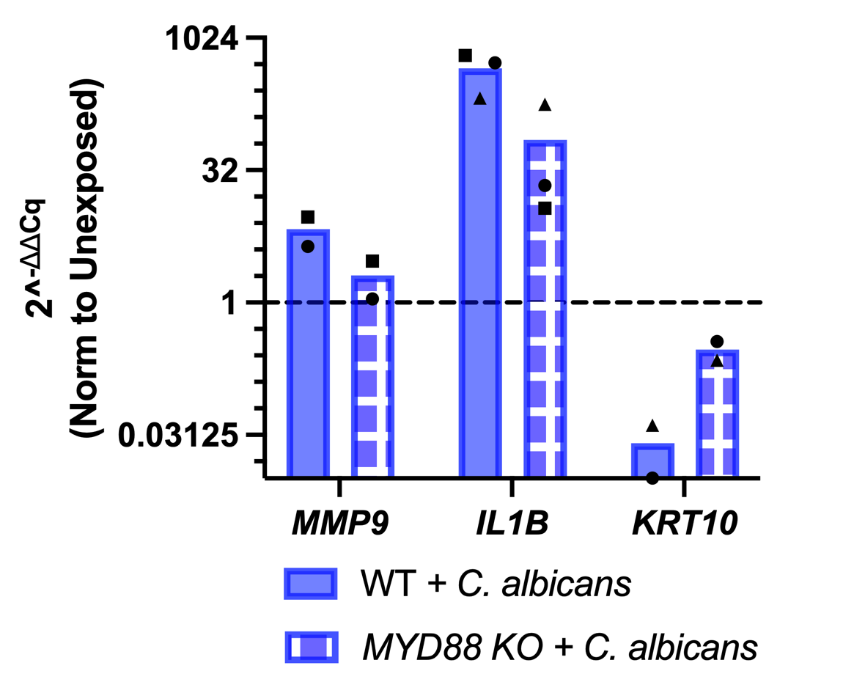


**Supplementary Figure 5. *MYD88*-Mediated regulation of *IL1B*, *MMP9* and *KRT10* transcription in KC.** RT-qPCR was performed to assess the relative expression of *IL1B*, *MMP9* and *KRT10* in WT (-gRNA) and *MYD88* KO KC after 24 hours of co-culture with *C. albicans*. Expression levels for genes of interest were normalized to the housekeeping gene *HPRT1* (*MMP9* & *KRT10*: *n*=2 experiments; *IL1B*: *n*=3 experiments).


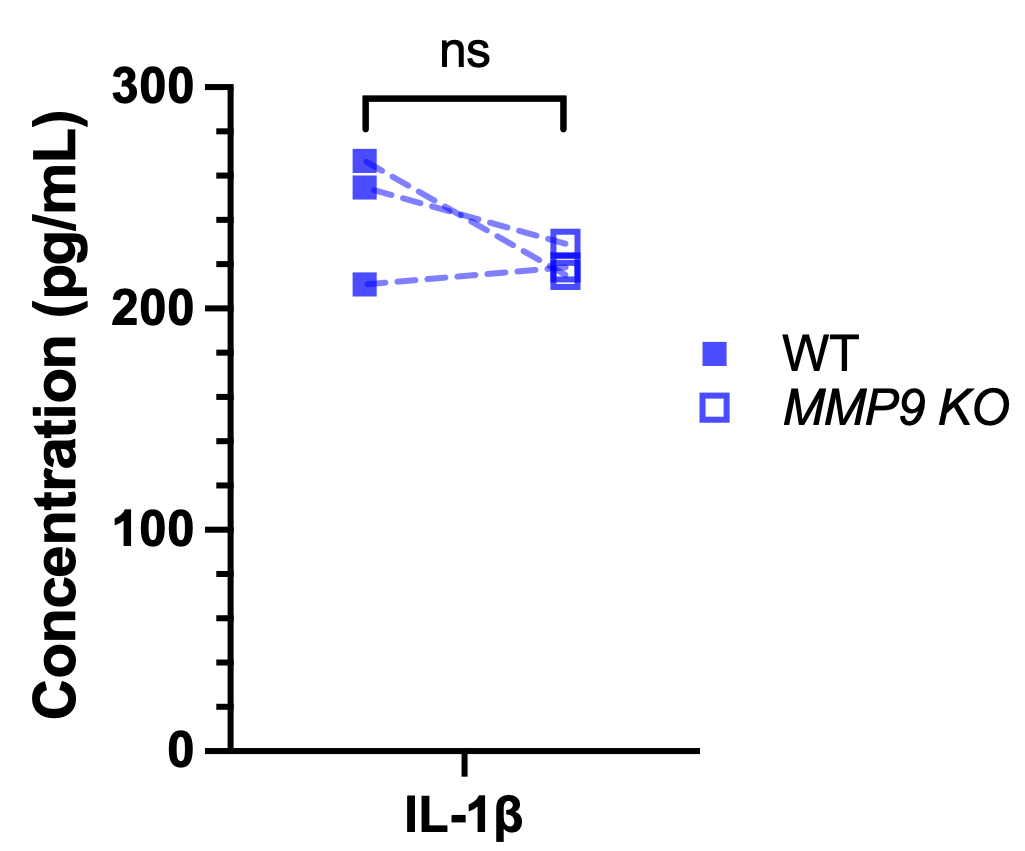


**Supplementary Figure 6.** **Loss of MMP-9 does not affect *C. albicans*-induced IL-1β secretion.** Quantification of secreted IL-1β in supernatants of WT and *MMP9* KO KC following 24 hours of *C. albicans* colonization (*n*=3 experiments).


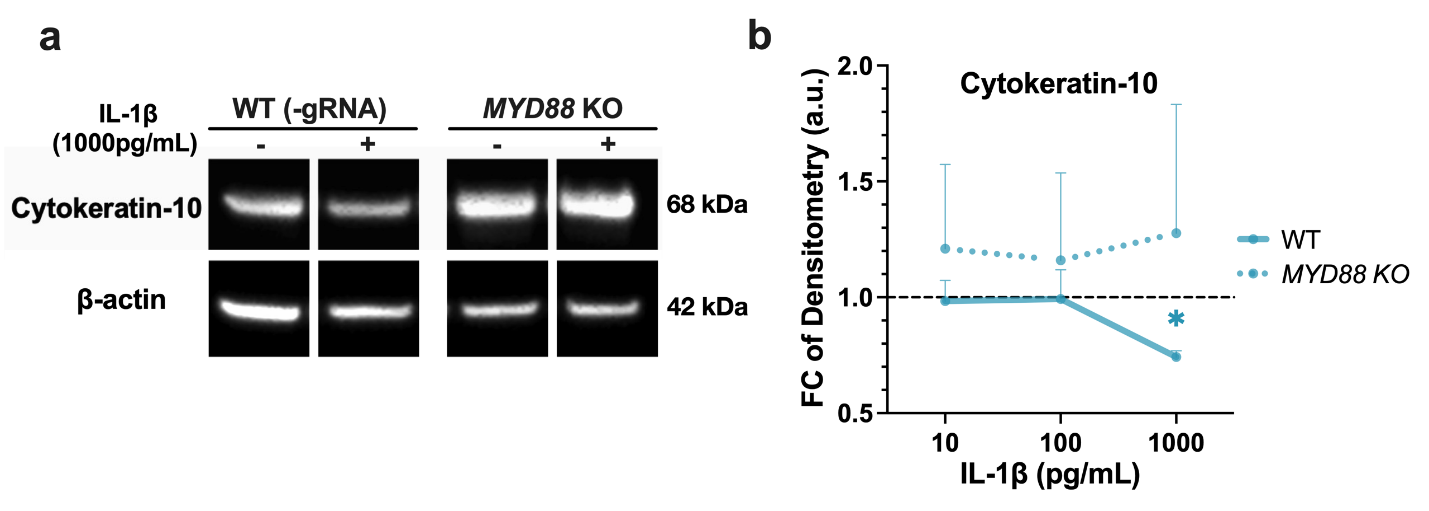


**Supplementary Figure 7. IL-1β treatment diminishes cytokeratin-10 expression through MyD88 signaling.** (a) Representative immunoblot showing cytokeratin-10 expression in WT and *MYD88* KO KC treated with IL-1β (10-1000 pg/mL) for 24 hours. (b) Quantification of cytokeratin-10 expression relative to untreated controls using densitometry (10 & 1000 pg/mL: *n*=3 experiments; 100 pg/mL: *n*=4 experiments). *p < 0.05.

| **Species** | **Isolate** |
| --- | --- |
| *Candida albicans* | SC5314 |
| *Candida albicans* | CAI4-Clp |
| *Candida albicans* | *sap1-3*∆/∆ |
| *Candida albicans* | *sap4-6*∆/∆ |
| *Candida albicans* | *ece1*∆/∆ |
| *Candida albicans* | URMLCA001 |
| *Candida albicans* | 25E31488 |
| *Candida albicans* | 25E31532 |
| *Candida albicans* | 25E31596 |
| *Candida parapsilosis* | 25E31469 |
| *Candida parapsilosis* | 25E31525 |
| *Candida parapsilosis* | 25E31576 |
| *Nakaseomyces glabratus* | URMLCG001 |
| *Nakaseomyces glabratus* | 25E31481 |
| *Nakaseomyces glabratus* | 25E31548 |
| *Nakaseomyces glabratus* | 25E31551 |
| *Candida tropicalis* | ATCC9968 |
| *Candida tropicalis* | 25E33144 |
| *Candida tropicalis* | 25E33458 |
| *Candida tropicalis* | 25E33465 |

**Supplementary Table 1**. Candida strains used in this study.

| Antibodies | Catalog #; Manufacturer | Dilution |
| --- | --- | --- |
| Anti-β-actin HRP (C4) | Cat # SC-47778 HRP; Santa Cruz Biotechnology | 1: 5000 |
| Anti-MMP9 (JA80-73) | Cat # MA5-32705; Invitrogen | 1: 5000 |
| Anti-cytokeratin 10 (Poly 19054) | Cat # 905403; BioLegend | 1: 2000 |
| Anti-rabbit IgG HRP (NA934V) | Cat # GENA934; Sigma-Aldrich | 1: 5000 |

Abbreviation: HRP, horseradish peroxidase

**Supplementary Table 2**. Antibodies used for western blot.

| Gene | Forward (5’- 3’) | Reverse (5’- 3’) |
| --- | --- | --- |
| *HPRT1* | TGC TGA GGA TTT GGA AAG GG | ACA GAG GGC TAC AAT GTG ATG |
| *IL1B* | GAT GGC TTA TTA CAG TGG CAA TGA | CAG AGG TCC AGG TCC TGG AA |
| *KRT10* | GGC AAA ATC AAG GAG TGG TAT G | TCG ATC TGA AGC AGG ATG TTG |
| *MMP9* | TGT ACC GCT ATG GTT ACA CTC G | GGC AGG GAC AGT TGC TTC T |
| *KLK7* | CCT GCT CAG TGG CAA TCA | GGT GCA CGG TGT ACT CAT TC |
| *TIMP1* | TCC AAG GCT CTG AAA AGG GC | ATT CAG GCT ATC TGG GAC CG |
| *CXCL8* | AAA CCA CCG GAA GGA ACC AT | GCT GCA GAA ATC AGG AGG GC |
| *IL6* | CTC TTC AGA ACG AAT TGA C | CTG CCA GTG CCT CTT T |
| *TNF* | AGG CAG TCA GAT CAT CTT CTC G | TCT TGA TGG CAG AGA GGA GG |

**Supplementary Table 3**. Primers used for RT-qPCR.
